# Mutual Information Gain and Linear/Nonlinear Redundancy for Agent Learning, Sequence Analysis, and Modeling

**DOI:** 10.3390/e22060608

**Published:** 2020-05-30

**Authors:** Jerry D. Gibson

**Affiliations:** Department of Electrical and Computer Engineering, University of California, Santa Barbara, CA 93106-9560, USA; gibson@ece.ucsb.edu

**Keywords:** agent learning, linear redundancy, nonlinear redundancy, mutual information gain

## Abstract

In many applications, intelligent agents need to identify any structure or apparent randomness in an environment and respond appropriately. We use the relative entropy to separate and quantify the presence of both linear and nonlinear redundancy in a sequence and we introduce the new quantities of total mutual information gain and incremental mutual information gain. We illustrate how these new quantities can be used to analyze and characterize the structures and apparent randomness for purely autoregressive sequences and for speech signals with long and short term linear redundancies. The mutual information gain is shown to be an important new tool for capturing and quantifying learning for sequence modeling and analysis.

## 1. Introduction

Many learning applications require agents to respond to their current environment for analysis or control. For these applications, agents need to either synchronize with and track the environment or at least have a good understanding of the current environment within which they are operating. Thus, one aspect of agent learning is concerned with discovering any structures in the environment, any changes in the structure of data sequences, and any randomness, however it may be defined, that may be present.

Analyses of learning with respect to identifying structures or changes in data sequences have often focussed on the classical Shannon entropy, its convergence to the entropy rate, and the relative entropy between subsequences, resulting in the definition of new quantities related to Shannon information theory that are defined to capture ideas relevant to these learning problems. Among these quantities are the terms entropy gain, information gain, redundancy, predictability, and excess entropy [1,2]. These newly defined quantities, while not necessarily new to classical information theoretic analyses, do yield insight into environmental behaviors and how a learning agent should operate within the given environment.

Although these information theoretic studies in agent learning have produced important insights into learning environments, there is still much more to be mined from Shannon information theory that can allow an agent to understand, track, synchronize, and operate within a perhaps changing environment. In this paper, we reexamine the fundamental quantity of relative entropy and consider the concepts of linear redundancy and nonlinear redundancy from lossy source coding and study the use of relative entropy for separating, discerning, and perhaps quantifying the presence of both linear redundancy and nonlinear redundancy in sequences.

These analyses lead to the definition of the new term, total redundancy, from which we obtain the new ideas of incremental mutual information gain and total mutual information gain. These new quantities allow a finer categorization of structure and randomness in sequences, thus admitting and facilitating new research directions and analyses. Our primary interest is in exploring relative entropy and the various related quantities for finite length sequences rather their asymptotic versions. The techniques used are variations on classical information theoretic quantities, and the novelty of the paper is in the introduction of new quantities, their applications, and new decompositions and insights, not in novel analysis tools.

Section 2 provides the needed background in information theory, most of which should be familiar, but with a few expressions that may not be commonly used. Section 3 covers entropy, entropy gain, information gain, redundancy, predictability, and excess entropy as commonly used in the agent learning literature. The concepts of linear and nonlinear redundancy from lossy source coding are introduced and developed from the viewpoint of agent learning in Section 4. Mutual information gain is defined and explored in Section 5, wherein the mutual information gain for Gaussian sequences is presented and the distribution free nature of mutual information gain is explained. Section 6 uses the prior quantities to address the modeling of autoregressive sequences and considers a specific purely autoregressive example. Speech signals, which are well represented by autoregressive models in some applications, but are more complex in that the order of the autoregressive model changes, there is often a longer term redundancy present, and the driving term is a mixed random and pseudo-periodic excitation, are analyzed in Section 7. Section 8 contains the conclusions.

## 2. Differential Entropy, Mutual Information, and Entropy Rate: Definitions and Notation

Given a continuous random variable *X* with probability density function p(x), the differential entropy is defined as

(1) $$h\left(X\right)=-\int_{-\infty }^{\infty }p(x)\log p(x)dx$$

where we assume *X* has the variance var(X) = $\sigma^{2}$. The differential entropy of a Gaussian sequence with mean zero and variance $\sigma^{2}$ is given by [3],

(2) $$h\left(X\right)=\frac{1}{2}\log2\pi e\sigma^{2}$$

An important quantity for investigating structure and randomness is the differential entropy rate [3]

(3) $$h\left(X\right)=\lim_{N\to \infty }\frac{1}{N}h\left(X_{1},\ldots ,X_{N}\right)$$

which is the long term average differential entropy in bits/symbol for the sequence being studied. For this paper, we use the differential entropy rate as an indicator of randomness. This is a simple indicator of randomness that has been used in similar agent learning papers [1,2].

For a stationary Gaussian process with (Toeplitz) correlation matrix $R_{N}$, its differential entropy is

(4) $$h\left(X_{1},X_{2},\ldots ,X_{n}\right)=\frac{1}{2}\log{\left(2\pi e\right)}^{n}\left|R_{N}\right|$$

with the corresponding differential entropy rate from Equation (3) given by

(5) $$h\left(X\right)=\frac{1}{2}\log2\pi e+\frac{1}{4\pi }\int_{-\pi }^{\pi }\log S(\lambda )d\lambda$$

where S(λ) is the power spectral density of the process.

An alternative definition of differential entropy rate is [3]

(6) $$h\left(X\right)=\lim_{N\to \infty }h\left(X_{N}|X_{N-1},\ldots ,X_{1}\right)$$

which for the Gaussian process yields

(7) $$h\left(X\right)=\frac{1}{2}\log2\pi e\sigma_{\infty }^{2}$$

where $\sigma_{\infty }^{2}$ is the minimum mean squared error of the best estimate given the infinite past, expressible as

(8) $$\sigma_{\infty }^{2}=\frac{1}{\left(2\pi e\right)}e^{2h(X)}\leq \sigma^{2}$$

with $\sigma^{2}$ and h(X) the variance and differential entropy rate of the original sequence, respectively.

Shannon gave the quantity $\sigma_{\infty }^{2}$ the notation *Q* and defined it to be the entropy power or entropy rate power, which is the power in a Gaussian process with the same differential entropy as the original random variable *X* [4]. Note that the original random variable or process does not need to be Gaussian. Whatever the form of h(X) for the original process, the entropy power can be defined as in Equation (8). In the following, we use h(X) for both differential entropy and differential entropy rate unless a clear distinction is needed to reduce confusion.

The differential entropy is defined for continuous amplitude random variables and processes, and it is the appropriate quantity to study signals such as speech, audio, and biological signals. However, unlike discrete entropy, differential entropy can be negative or infinite, and is changed by scaling and similar transformations. Note that this is why mutual information is often the better choice for investigating learning applications.

To translate differential entropy into a useful indicator when considered alone, it is necessary to use a result from Cover and Thomas [3] that, for a continuous random variable *X*, the discrete entropy in terms of the differential entropy is

(9) H(X)≈h(X)+n

where *n* is the number of bits used in the quantization of the random variable *X*. Note that this is the same expression obtained for the discrete entropy of the quantizer output for high rate scalar quantization subject to a mean squared error distortion measure for an input with differential entropy h(X) [5]. For a Gaussian random variable with zero mean and variance $\sigma^{2}$, then Equation (9) becomes

(10) $$H\left(X\right)\approx n+\frac{1}{2}\log2\pi e\sigma^{2}$$

We use this result in later examples.

A useful and commonly used measure of the distance between two probability distributions p(x) and q(x), x∈X is the relative entropy or Kullback–Leibler divergence defined as [3]

(11) $$D\left(p\|q\right)=\int p\left(x\right)\log\frac{p(x)}{q(x)}dx$$

A special case of the relative entropy is the mutual information. For continuous random variables *X* and *Y* with probability density functions p(x) and p(y), respectively, the mutual information between *X* and *Y*

(12) I(X;Y)=h(X)−h(X|Y)=h(Y)−h(Y|X)

Given the continuous random variables $X_{1},X_{2},\ldots ,X_{n}$, and *Y*, the chain rule for mutual information is

(13) $$\begin{array}{cc} I(X_{1},X_{2},\ldots ,X_{n};Y)= & h\left(X_{1},X_{2},\ldots ,X_{n}\right)-h\left(X_{1},X_{2},\ldots ,X_{n}|Y\right)\\ = & \sum_{i=1}^{n}I\left(X_{i};Y|X_{1},X_{2},\ldots ,X_{i-1}\right) \end{array}$$

To separate structure and apparent randomness in sequences, consider *n* successive values of the sequence $X^{\left(n\right)}=\left(x_{1},x_{2},\ldots ,x_{n}\right)$, and examine the relative entropy of the joint probability density of this sequence $P_{X}^{\left(n\right)}\left(X\right)=P_{X}^{\left(n\right)}\left(x_{1},x_{2},\ldots ,x_{n}\right)$ with respect to a memoryless sequence $X^{*}$ that has the same product of the first order marginal densities, $P_{X^{*}}^{\left(n\right)}\left(X^{*}\right)=P_{X^{*}}^{\left(n\right)}\left(x_{1},x_{2},\ldots ,x_{n}\right)=\prod_{k=1}^{n}P_{X}^{\left(1\right)}\left(x_{k}\right)$ so

(14) $$D_{n}(P_{X}^{\left(n\right)}\left(X\right)\|P_{X^{*}}^{\left(n\right)}\left(X^{*}\right))=\frac{1}{n}\int P_{X}^{\left(n\right)}\left(x\right)\log\frac{P_{X}^{\left(n\right)}\left(x\right)}{P_{X^{*}}^{\left(n\right)}\left(x^{*}\right)}dx^{n}$$

This straightforward quantity is useful since what we need is an indicator of change between two situations; that is, if we calculate the relative entropy in Equation (14) before we do some processing or transformation and afterward, does the relative entropy capture a relative change?

Another type of randomness of interest is the relationship of the i.i.d. density of a sequence with respect to an uniform distribution. This relationship can be captured by the relative entropy between the product of first order marginal densities of a sequence and an uniform distribution as

(15) $$D_{n}(\prod_{k=1}^{n}P_{X}^{\left(1\right)}\left(x_{k}\right)\|U^{\left(n\right)})=\frac{1}{n}\int P_{X^{*}}^{\left(n\right)}\left(x^{*}\right)\log\frac{P_{X^{*}}^{\left(n\right)}\left(x^{*}\right)}{U^{\left(n\right)}\left(x\right)}dx^{n}$$

The relative entropy of the joint distribution with respect to a uniform distribution is also of interest in learning problems and this relative entropy can be expressed as the sum of the relative entropies in Equations (14) and (15) as

(16) $$D_{n}(P_{X}^{\left(n\right)}\left(X\right)\|U^{\left(n\right)})=D_{n}(P_{X}^{\left(n\right)}\left(X\right)\|\prod_{k=1}^{n}P_{X}^{\left(1\right)}\left(x_{k}\right))+D_{n}(\prod_{k=1}^{n}P_{X}^{\left(1\right)}\left(x_{k}\right)\|U^{\left(n\right)})$$

by using a chain rule for relative entropy [3].

The relative entropy is prevalent in agent learning analyses as is shown in the following section. The expressions for relative entropy in Equations (14)–(16), although straightforward, allow deeper insights into existing structure and apparent randomness in sequences, and examples are provided in later sections of what these expressions reveal.

## 3. Agent Learning and Redundancy

In reinforcement learning, the goal (broadly) is to observe the environment, understand the behavior of the environment, and then take action to operate successfully within that environment. Our focus in this paper is on the agent learning component wherein upon taking some observations of the environment, we develop an understanding of the structure of the environment, formulate models of this structure, and study any remaining apparent randomness or unpredictability.

Results from agent learning have made use of the information theoretic ideas in Section 2, and have created variations on those information theoretic ideas to capture particular characteristics that are distinct to agent learning problems. We summarize a few of these variations and newly defined quantities here.

In the agent learning literature, it is desired to explore the broad ideas of unpredictability and apparent randomness [1,2]. Toward this end, it is common to investigate the total Shannon entropy of length-*N* sequences given by

(17) $$h\left(X^{\left(N\right)}\right)=-\int P_{X}^{\left(N\right)}\left(X\right)\log P_{X}^{\left(N\right)}\left(X\right)dX^{N}$$

as a function of *N* to characterize learning. The name total Shannon entropy is appropriate since it is not the usual per component entropy of interest in lossless source coding [3], for example.

In association with the idea of learning or discerning structure in an environment, the entropy gain is defined as the difference between the entropies of length *N* and length N−1 sequences as [2]

(18) $$\Delta H\left(N\right)=h\left(X^{N}\right)-h\left(X^{N-1}\right)$$

Equation (18) was derived and studied much earlier by Shannon [4] not as an entropy gain but as a conditional entropy.

In particular, Shannon [4] defined the conditional entropy of the next symbol when the N−1 preceding symbols are known as

(19) $$h\left(X_{N}|X^{N-1}\right)=h\left(X_{N},X^{N-1}\right)-h\left(X^{N-1}\right)=h\left(X^{N}\right)-h\left(X^{N-1}\right)$$

which is exactly Equation (18); so the entropy gain from the agent learning literature is simply the conditional entropy expression developed by Shannon in 1948.

Elias [6] considered the conditional entropy introduced by Shannon and called it the entropy added by the Nth term, which again is consistent with the designation of entropy gain in the agent learning literature as in Equation (18). Elias desired to find an upper bound on this added entropy. Noting that the differential entropy of an Nth order Gaussian sequence is given by $\frac{1}{2}\log\left[2\pi e|R_{N}{|}^{1/N}\right]$, Elias shows that the entropy added by the Nth term is

(20) $$\Delta H\left(N\right)=\frac{1}{2}\log2\pi e\frac{|R_{N+1}|}{|R_{N}|}$$

Going beyond the concept of entropy gain, a definition of information gain, represented by ΔH(N) and expressed as a relative entropy has been offered and studied by Crutchfield and Feldman [1,2] as

(21) $$\Delta H\left(N\right)=D(P_{X}^{\left(N\right)}\left(X\right)\|P_{X}^{\left(N-1\right)}\left(X\right))$$

In Equation (21), the support set of the two distributions is not the same, so the $P_{X}^{\left(N-1\right)}\left(X\right)$ is extended by concatenating all values of the $x_{N}$ symbol with the prior symbols $x_{0},x_{1},\ldots ,x_{N-1}$ with equal probability [2].

It is also shown in [2] that (this result is in Shannon [4] and Elias [6] as well)

(22) $$\bar{h}=\lim_{N\to \infty }\Delta H\left(N\right)$$

which is the definition of differential entropy rate stated in Equation (3), and where we let $\bar{h}=h\left(X\right)$ for notational compactness and to be consistent with [2].

Further, in the learning literature, two definitions of a quantity called redundancy are offered. One definition is as the difference between the maximum value of the entropy rate log|X|, where |X| is the cardinality of a discrete alphabet or the volume of the support set for a continuous variable, and the entropy rate $\bar{h}$ so that the redundancy is [2]

(23) $$R=\log|X|-\bar{h}$$

A second definition of redundancy $D_{N}(P_{X}^{\left(N\right)}\left(X\right)||U^{\left(N\right)})$ is the relative entropy between the known distribution $P_{X}^{\left(N\right)}$ and the uniform distribution, $U^{\left(N\right)}$, asymptotically in *N*,

(24) $$R=\lim_{N\to \infty }D_{N}(P_{X}^{\left(N\right)}\left(X\right)\|U^{\left(N\right)})$$

Thus, for the definitions of redundancy in Equations (23) and (24), it can be stated that the redundancy R is an indicator of the information gained when an agent learns that the actual distribution is different from an uniform distribution as the sequence length becomes asymptotically large.

To study how the redundancy evolves with finite length *N* observations of the environment, a version of the redundancy, called *N*-*redundancy*, is defined if the actual distribution of the length *N* sequence is known to be $P_{X}^{\left(N\right)}$, so the entropy is $h(X_{1},\ldots ,X_{N})$ and [2]

(25) $$R\left(N\right)\equiv h\left(X_{1},\ldots ,X_{N}\right)-N\bar{h}$$

Equations (24) and (25) are special cases of the generalized definition of redundancy from information theory which is the difference between the expected length of a lossless code and the lower limit for the expected length of the code, expressed in terms of a relative entropy [3].

A characterization of the per symbol entropy when *N* observations of the environment are available compared to the per symbol entropy with an infinite number of measurements is given by the per symbol *N*-redundancy defined as

(26) $$r\left(N\right)=\Delta R\left(N\right)=\Delta H\left(N\right)-\bar{h}$$

The quantity r(N) has also been called the local or *N*-dependent predictability [7].

To capture the total amount of redundancy per symbol as a measure of memory in an environment, Crutchfield and Feldman [1] define the quantity Excess Entropy as

(27) $$E=\lim_{N\to \infty }R\left(N\right)\equiv \lim_{N\to \infty }\left[h\left(X_{1},\ldots ,X_{N}\right)-N\bar{h}\right]$$

which is the limit of the redundancy in Equation (25). We contrast our results with the excess entropy in later examples.

The entropy and the differential entropy rate are the primary workhorses in agent learning analyses related to reinforcement learning and curiosity learning scenarios [1,2]. As a result, the definitions of information gain and redundancy from the agent learning literature as presented in this current section are perhaps too expansive and too imprecise in several ways and should be, and can be, refined to allow the observation of additional phenomena.

In the following section we provide definitions of the new quantities, linear redundancy and nonlinear redundancy and mutual information gain, that are more in line with Shannon theory and also allow more detailed parsing of what is happening in the learning process.

## 4. Linear and Nonlinear Redundancy

Some definitions of redundancy and predictability from the information theoretic lossy source coding literature allow the redundancy in a sequence to be broken down further than with the definitions in Section 3. In lossy source coding, it is recognized that two types of redundancy can be defined, namely, linear redundancy and nonlinear redundancy. The former is sometimes called correlation redundancy, and is often used in linear prediction, and the latter is often called statistical redundancy, which captures the statistical dependence between quantities when the linear redundancy (linear predictability) is removed [8].

The relative entropy in Equation (14) can be associated with the linear redundancy, denoted as $R_{lin}$,

(28) $$R_{lin}=D_{n}(P_{X}^{\left(n\right)}\left(X\right)\|\prod_{k=1}^{n}P_{X}^{\left(1\right)}\left(x_{k}\right))$$

which captures the memory with respect to an i.i.d. version of the sequence.

The relative entropy in Equation (15) can be associated with the nonlinear redundancy, denoted as $R_{non}$,

(29) $$R_{non}=D_{n}(\prod_{k=1}^{n}P_{X}^{\left(1\right)}\left(x_{k}\right)\|U^{\left(n\right)})$$

expresses the relative entropy of an i.i.d. sequence formed from the marginals of the sequence with respect to a uniform distribution.

Splitting the redundancy into linear and nonlinear components as in Equations (28) and (29) is apparently new, particularly in the learning literature; further, splitting the redundancy into the linear and nonlinear components allows the exploration of structure in the sequence in finer detail, which is particularly useful when developing models for the sequence being explored.

A useful variation on Equation (28) is to limit the memory of the sequence that is observable. In particular, consider the relative entropy involving only the current and immediate past *M* samples of the sequence, denoted as the *M*-redundancy, given by

(30) $$R_{linM}=D(p\left(X_{N},\ldots ,X_{N-M}\right)\|p\left(X_{N}\right)\cdots p\left(X_{N-M}\right))$$

which is the linear redundancy with respect to a finite past history. Notationally, letting $X^{N-M}=X_{N-M},\ldots ,X_{N-1}$, the *M*-redundancy in Equation (30) can be expanded as

(31) $$\begin{array}{cc} R_{linM}= & D(p\left(X_{N},X^{N-M}\right)\|p\left(X_{N}\right)\cdots p\left(X_{N-M}\right))\\ = & I\left(X_{N};X^{N-M}\right)+h\left(X^{N-M}\right)+D(p\left(X_{N-1},\ldots ,X_{N-M}\right)\|p\left(X_{N-1}\right)\cdots p\left(X_{N-M}\right)) \end{array}$$

The last term in Equation (31) is

(32) $$D(p\left(X_{N-1},\ldots ,X_{N-M}\right)\|p\left(X_{N-1}\right)\cdots p\left(X_{N-M}\right))=-h\left(X^{N-M}\right)+\sum_{i=N-M}^{N-1}h\left(X_{i}\right)$$

so the *M*-redundancy in Equation (31) takes the simple form

(33) $$R_{linM}=I\left(X_{N};X^{N-M}\right)+\sum_{i=N-M}^{N-1}h\left(X_{i}\right)=I\left(X_{N};X^{N-M}\right)+Mh\left(X\right)$$

where the first equality follows from independence and the last follows if there is stationarity. Thus, the *M*-redundancy equals the mutual information between the current sample of the sequence and the immediate past *M* samples plus the sum of the differential entropies of the past *M* values of the random sequence.

The nonlinear redundancy can also be simplified. To do this, the uniform distribution is assumed to have wide but finite support and the number of quantization levels $L=2^{n}$ are assumed sufficient that the probability density over the support is $U^{\left(n\right)}=2^{-nM}$ for the *M* samples. Therefore,

(34) $$D(p\left(X_{N-1},X_{N-2},\ldots ,X_{N-M}\right)\left|\right|2^{-nM})=-\sum_{i=N-M}^{N-1}h\left(X_{i}\right)+nM$$

Using Equations (33) and (34) in Equation (16) yields

(35) $$R_{T}=D(p\left(X_{N},X^{N-M}\right)\|2^{-nM})=I\left(X_{N};X^{N-M}\right)+nM$$

for the total redundancy. However, it is the decomposition of the redundancy into linear and nonlinear redundancy that opens the door to some new insights and useful new analyses.

There might be two time scales for the linear redundancy so a further decomposition of the linear redundancy into long term and short term redundancies may be useful in many applications and such an analysis is provided in a later section, Section 7, on speech processing.

As we have seen in prior sections and as will be developed subsequently, the separation of these two redundancies/predictabilities can provide (and have provided) different insights into learning and modeling for signal processing.

## 5. Mutual Information Gain

Even though Equation (21) has been called information gain in the agent learning literature, it is clear from Equations (18) and (19) that it is a conditional entropy. As such, the nomenclature, information gain, is misleading. In terms of information gain, as can be seen from Equation (35), the quantity of interest is the mutual information between the overall sequence and the growing history of the past given by

(36) $$\begin{array}{cc} I(X_{N};X^{N-1}) & =h\left(X_{N}\right)-h\left(X_{N}|X^{N-1}\right)\\ \; & =h\left(X_{N}\right)-\left[h\left(X^{N}\right)-h\left(X^{N-1}\right)\right]\\ \; & =h\left(X_{N}\right)-\Delta H\left(N\right) \end{array}$$

where ΔH(N) is defined in Equation (18). The mutual information in Equation (36) is much more intuitive as a measure of information gained as a a function of *N* and includes the entropy gain from agent learning as a natural component.

We can obtain more insight by expanding Equation (36) using the chain rule for mutual information in Equation (13) [3] as

(37) $$\begin{array}{cc} I(X_{N};X^{N-1}) & =h\left(X_{N}\right)-h\left(X_{N}|X^{N-1}\right)=\sum_{k=1}^{N-1}I\left(X_{N};X_{k}|X_{k-1},\ldots ,X_{0}\right)\\ \; & =I\left(X_{N};X_{N-1}|X_{N-2},\ldots ,X_{1},X_{0}\right)+\ldots +I\left(X_{N};X_{2}|X_{1},X_{0}\right)+I\left(X_{N};X_{1}|X_{0}\right) \end{array}$$

Since $I(X_{N};X_{k-1}|X_{k-2},\ldots ,X_{0})\geq 0$, we see that $I(X_{N};X^{N-1})$ is nondecreasing in *N*; however, what do these individual terms in Equation (37) mean? The sequence $X_{N}$ should be considered the input sequence to be analyzed with the block length *N* large but finite. The first term in the sum, $I(X_{N};X_{1}|X_{0})$ indicates the mutual information between the predicted value of $X_{1}$, given $X_{0}$, and the input sequence $X_{N}$. The next term $I(X_{N};X_{2}|X_{1},X_{0})$ is the mutual information between the input sequence $X_{N}$ and the predicted value of $X_{2}$, given the prior values $X_{1},X_{0}$. Therefore, we can characterize the change in mutual information with increasing knowledge of the past history of the sequence as a sum of conditional mutual informations $I(X_{N};X_{k-1}|X_{k-2},\ldots ,X_{0})$.

We denote $I(X_{N};X^{N-1})$ as the total mutual information gain and $I(X_{N};X_{k-1}|X_{k-2},\ldots ,X_{0})$ as the incremental mutual information gain. Clearly, there are substantive differences between the information gain as defined in Equation (21), which is really only an entropy gain expressed as a relative entropy, and the new concepts of total mutual information gain and incremental mutual information gain in terms of mutual informations.

We can also consider the mutual information between the input sequence $X_{N}$ and the immediate past values $X_{N-M}$, M<N, which is

(38) $$\begin{array}{cc} I(X_{N};X^{N-M}) & =h\left(X_{N}\right)-h\left(X_{N}|X^{N-M}\right)=\sum_{k=1}^{M}I\left(X_{N};X_{N-k}|X_{N-k-1},\ldots ,X_{N-M}\right)\\ = & I\left(X_{N};X_{N-1}|X_{N-2},\ldots ,X_{N-M}\right)+\ldots +I\left(X_{N};X_{N-M-1}|X_{N-M}\right)+I\left(X_{N};X_{N-M}\right) \end{array}$$

This expression allows the input block length *N* to be finite if we need it to be so and it also allows the past history *M* to be finite, which may occur due to having a finite memory for the analyses.

Thus, the definitions of entropy gain in Equations (18) and (21) are now distinct from the mutual information gain in Equations (37) and (38), as is desirable.

### 5.1. Stationary and Gaussian

We can say more if the sequence $X_{k}$ is stationary and Gaussian with $EX_{k}=0$, $EX_{k}X_{k+n}=\rho_{n}$, and $EX_{k}^{2}=\sigma^{2}$. Then, we know that

(39) $$h\left(X_{N}|X^{M-1}\right)=\frac{1}{2}\log2\pi eMMSPE(M)$$

with $MMSPE\left(M\right)=\frac{|R_{M+1}|}{|R_{M}|}$, where the matrices are populated with the $\rho_{n}$ terms. With stationary and independent $X_{k}$, then $h\left(X_{N}\right)=h\left(X\right)=\frac{1}{2}\log2\pi e\sigma^{2}$, so using Equations (20) and (38), we find that

(40) $$I\left(X_{N};X^{N-M}\right)=h\left(X_{N}\right)-h\left(X_{N}|X^{N-M}\right)=\frac{1}{2}\log\frac{\sigma^{2}}{MMSPE(M)}$$

This is an important expression for the mutual information gain since knowing the sequence variance and the minimum mean squared prediction error for an Mth order predictor, we can evaluate total mutual information gain without having to approximate the probability distributions and the entropies.

The utility of the mutual information gain expressions in Equations (37) and (38) becomes even more evident under the Gaussian assumption since the conditional mutual information terms become

(41) $$\begin{array}{cc} I(X_{N};X_{k}|X_{k-1},\ldots ,X_{N-M})= & h\left(X_{N}|X_{k-1},\ldots ,X_{N-M}\right)-h\left(X_{N}|X_{k},X_{k-1},\ldots ,X_{N-M}\right)\\ = & \frac{1}{2}\log\frac{\sigma_{e(k-1)}^{2}}{\sigma_{ek}^{2}} \end{array}$$

Then we have for Equation (38)

(42) $$\begin{array}{cc} \; & I\left(X_{N};X^{N-M}\right)=h\left(X_{N}\right)-h\left(X_{N}|X^{N-M}\right)\\ \; & =\frac{1}{2}\left[\log\frac{\sigma^{2}}{\sigma_{e1}^{2}}+\log\frac{\sigma_{e1}^{2}}{\sigma_{e2}^{2}}\cdots +\log\frac{\sigma_{e(N-M-1)}^{2}}{\sigma_{e(N-M)}^{2}}\right] \end{array}$$

We know that the minimum mean squared prediction error is nonincreasing $\sigma_{e(n-1)}^{2}\geq \sigma_{e(n)}^{2}$, so each term in the sum in Equation (42) is greater than or equal to zero, as must be true since it is a mutual information.

We see from Equations (38), (40), and (42) that the mutual information gain gives us a quantitative indicator in bits/symbol of the linear redundancy being captured or modeled. This is a new and useful indicator of structure or memory being separated from randomness.

### 5.2. A Distribution Free Information Measure

If we compare the mutual information in Equation (40) with the entropy gain expression in Equation (20), the scaling factor for the Gaussian density has been divided out and is not present in Equation (40). This lack of scaling is important when interpreting the mutual information gain since it is no longer dependent on the underlying distribution that would create a bias term.

Note that the prior definition of information gain in agent learning in Equation (21) is actually an entropy gain so the scaling factor is present. The new quantity, total mutual information gain, therefore, has a distribution free property not satisfied by entropy gain. In fact, for continuous random variables, the differential entropy can be changed by a linear transformation but the mutual information cannot [3].

## 6. Autoregressive Modeling

An autoregessive (AR) process is given by

(43) $$x\left(k\right)=\sum_{i=1}^{M}a_{i}x\left(k-i\right)+w\left(k\right)$$

where the $a_{i},i=1,2,\ldots M$ are called autoregressive parameters and w(k) is the excitation sequence. Let us assume that the sequence being analyzed is a stationary, purely autoregressive sequence of order *M* and the excitation term w(k) has the possibly nonuniform probability density function $p_{W}\left(w\right)$ with variance $\sigma^{2}$.

We can then use Equation (35) to expand the redundancy for this sequence. This makes explicit the fact that the distance from randomness consists of two components, the linear redundancy due to the predictive component and the nonlinear redundancy due to the distribution of the excitation.

If we know the true autoregressive parameters and the correct AR model order for a sequence, then the linear redundancy can be removed by operating on the given sequence so that the remaining distance from randomness is the nonlinear redundancy only. However, in most learning and modeling problems, even if we are willing to assume that the sequence being observed is autoregressive, the true AR model order is not known. The following example explores these ideas.

### Example: Learning and Modeling an AR Sequence

A zero mean unit variance purely AR(10) Gaussian sequence is given by Equation (43) with coefficients $a_{1}=2.0965,a_{2}=-2.6235,a_{3}=1.4123,a_{4}=-0.8282,a_{5}=0.5066,a_{6}=-0.1511,a_{7}=-0.7505,a_{8}=1.1628,a_{9}=-0.7748,a_{10}=0.1906$, where the sequence w(k) is Gaussian with zero mean and variance $\sigma_{W}^{2}$. (Note that these autoregressive parameters, $a_{i},i=1,2,\ldots M$, were obtained by processing a frame of speech sampled at 8000 samples/sec to calculate the autocorrelation terms and then using the techniques in Appendix A.) Table 1 shows the incremental mutual information gain and the total mutual information gain as the predictor order *M* is increased.

We observe that the MMSPE ($\sigma_{e(M)}^{2}$) is decreasing monotonically but not so for the incremental mutual information gain, which increases in going from a 1st order predictor to a 2nd order predictor and also in going from a 3rd order predictor to an M=4th order predictor and further when the predictor order goes from M=8 to M=9. Perhaps this hints at why mean squared error is thought not to be a reliable indicator of performance in learning applications.

**Figure 1:**
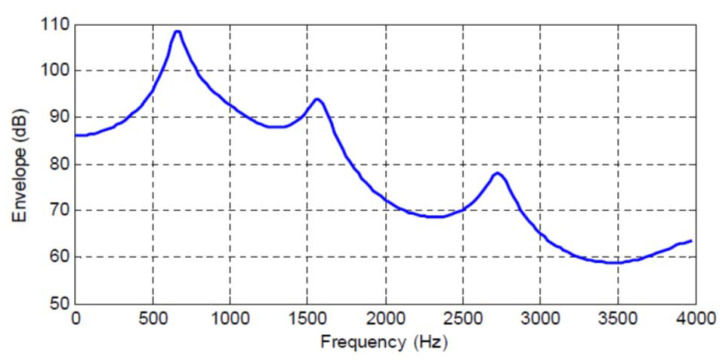
Autoregressive (AR) model frequency response.

However, there is an even tighter connection between these increases in mutual information gain and the physical process inherent in the autoregressive model with the given coefficients. The frequency response corresponding to the AR model in Equation (43) and the given coefficients is plotted in Figure 1. There are three major peaks evident in the spectrum, but certainly the relative magnitudes of the peaks are quite different. As noted from Table 1, there are jumps in the incremental mutual information gain was the predictor order changes from 0 to 1, from 1 to 2, from 3 to 4, and from 8 to 9. There is a general rule that to represent a peak in a spectral envelope requires two model coefficients, which when translated to the frequency domain provide the location of the spectral peak and the bandwidth of that peak.

The increase in predictor order from 0 to 2 corresponds to representing the first spectral peak in Figure 1, the increase from 3 to 4 would allow us to capture the information due to the second spectral peak, and the increase from 8 to 9 indicates the third spectral peak. If we were to plot the spectra as the predictor order is increased from 0 to 10, this evolution would be clearer with the substantial jump in incremental mutual information gain in going from 0 to 1 showing a magnitude at low frequencies and rough location of the peak but not the bandwidth (not an isolated peak itself). Further discussion of these ideas are more properly in the context of time series analysis or linear prediction of speech [9] than in the present development of this example; however, it is evident that the incremental mutual information gain indicates significant physical changes in the underlying sequence that, while present in the changes in mean squared prediction error, they are not highlighted as with the incremental mutual information gain.

The total mutual information gain 0f 2.647 bits/symbol is the gain that comes from the linear redundancy in the AR(10) sequence, and the remaining redundancy is the nonlinear redundancy. If this sequence is modeled with a M=2nd order predictor, that is, if the AR(10) sequence is modeled as an AR(2) sequence, we would conclude that the mutual information gain or linear redundancy of such a sequence was only 1.952 bits/symbol with $\sigma_{W}^{2}=\sigma_{e(2)}^{2}=0.0667$.

The redundancy not captured by the prediction, as represented by the $\sigma_{e(2)}^{2}$ value would be associated with nonlinear redundancy. The driving term w(k) would then be considered a spectrally white process and the entropy rate associated with the nonlinear redundancy would be given by Equation (5) with $S\left(\lambda \right)=\sigma_{e(2)}^{2}$ for −π≤λ≤π, so from Equation (10), the entropy rate would erroneously be thought to be $h\left(X\right)=\frac{1}{2}\log2\pi e\sigma_{e(2)}^{2}$.

From Equation (10), the entropy (discrete) in the nonlinear redundancy for the predictor order M=10 is

(44) $$H\left(E\right)\approx n+\frac{1}{2}\log2\pi e\sigma_{\infty }^{2}=n-0.611\;\mathrm{bits}/\mathrm{symbol}$$

where we have used the notation *E* for the prediction error random variable.

Note that we can verify the result that the total mutual information gain given in Table 1 for M=10 is reasonable by noting that the discrete entropy associated with the original AR(10) sequence before the removal of the linear redundancy is $H\left(X\right)\approx n+\frac{1}{2}\log2\pi e(1)$ since $\sigma^{2}=1$. Thus, H(X)≈n+2.047 bits/ symbol. The total change in differential entropy is therefore H(X)−H(E)≈2.658 bits/symbol, which closely agrees with the total mutual information gain of 2.647 bits/symbol.

The important step of using mutual information gain rather than the differential entropy removes the need to consider the projection of the differential entropy back into a discrete entropy, and yields a quantity that stands alone both incrementally and as a total. Furthermore, the mutual information gain is more sensitive to what is actually happening in the learning process. More explicitly, for M=2 in the example, $\sigma_{e(2)}^{2}=0.0667$ whereas for M=10, the mean squared prediction error is $\sigma_{e(10)}^{2}=\sigma_{\infty }^{2}=0.0261$, which does not appear to be much different from M=2. However, the difference in mutual information gain is 2.647−1.952=0.695 bits/symbol. So mutual information gain is a more sensitive indicator of performance in learning applications.

As illustrated in the example, if we utilize the incorrect AR model order L<M and assume it is correct, we associate the excess unmodeled linear redundancy with the nonlinear redundancy, thus getting a misleading interpretation of the amount of randomness in the sequence we are observing. Other modeling errors, such as incorrect AR model coefficients or a sequence that is only partially autoregressive, will invite similar incorrect conclusions about the character of the sequence being analyzed or modeled.

Crutchfield and Feldman [1] study the same phenomenon of unmodeled structure in sequences using the quantity, Excess Entropy, as defined in Equation (27). The separation of redundancy into linear and nonlinear redundancy as discussed in Section 4 and shown in Equations (30) and (34) allows greater insight into the sources of unmodeled randomness than the quantity of excess entropy alone, which does not separate out the linear and nonlinear redundancies.

In the following section, we address the analysis of speech signals using the ideas of linear and nonlinear redundancy and mutual information gain. Speech is well-suited to such a study since it is known to be well-modeled by the AR model in many instances, but not always, and further, even when the AR model is useful, as in speech coding, the model order is not known precisely and there are different types of sounds, such as nasal sounds, that are not accurately produced by the AR model.

## 7. Speech Processing

Speech is an interesting and important signal for which AR modeling, called the linear prediction model, has had extraordinary success for speech coding and other speech processing applications [9,10,11], however, speech is not a purely AR sequence, and further the model order and the coefficients are not known exactly. As a consequence, there are several unmodeled components that may appear as nonlinear redundancy and thus can cause the distance from randomness to appear larger than it is. Therefore, the application of our results to speech analysis is especially interesting, given the importance of speech applications and the challenging analysis.

We begin with fitting a 10th order AR model to a speech segment. The chosen model order of M=10 agrees with what is often assumed in speech coding applications, but need not be the true or best model order for any particular speech segment. We do not know the AR model coefficients so we have to calculate them.

### 7.1. AR Speech Model

For these analyses to explore the ideas of linear and nonlinear redundancy and mutual information gain, we utilize a block approach to calculating the AR model parameters. However, in agent learning applications as we envision here, it may be more natural to employ a sequential or recursive algorithm that processes the speech sequence in a sample-by-sample manner. The recursive algorithms are less common in speech analyses and appear more complicated than the block approach, so we use the block approach to illustrate the application and insights provided by the new terms linear redundancy, nonlinear redundancy, and mutual information gain. However, the recursive algorithms can also be used for AR model analyses and prediction.

**Figure 2:**
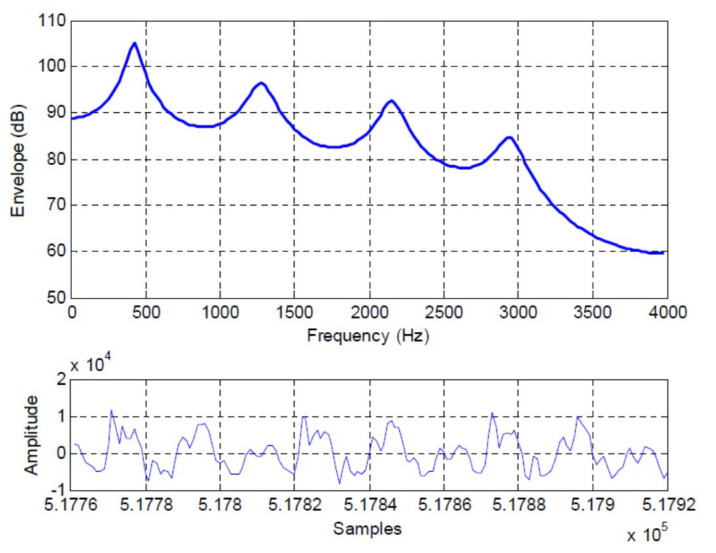
Frame 3237 time domain waveform (bottom) and spectral envelope, SPER = 9.15 dB.

As a specific example, we analyze the 160 time domain samples (bandlimited to 3400 Hz and sampled 8000 samples/sec) plotted at the bottom of Figure 2, which has the normalized autocorrelation terms R(0)=1.0,R(1)=0.7728,R(2)=0.4242,R(3)=0.2425,R(4)=0.1002, R(5)=−0.0461, R(6)=−0.1608,R(7)=−0.2799,R(8)=−0.4802,R(9)=−0.6799, and R(10)=−0.6344. The spectral envelope of this frame is shown in the top portion of the figure. The AR coefficients are calculated as indicated in Appendix A.

The MMSPE(M) values as the AR model order *M* is increased from 1 on up to 10 are shown on the left side of Table 2. The middle column, labeled $I(X_{N};X_{M}|X_{M-1},\ldots )$, contains the incremental mutual information gain as the predictor order is increased. The rightmost column is the total mutual information gain for that model order, which is given by

(45) $$\begin{array}{cc} \; & I\left(X_{N};X^{N-M}\right)=h\left(X_{N}\right)-h\left(X_{N}|X^{N-M}\right)\\ \; & =\frac{1}{2}\left[\log\frac{\sigma^{2}}{\sigma_{e1}^{2}}+\sum_{i=1}^{M-1}\left(\log\frac{\sigma_{ei}^{2}}{\sigma_{e(i+1)}^{2}}\right)\right] \end{array}$$

for M≥2, and which simplifies to just the first term for M=1.

From Table 2, we see that the MMSPE(M), denoted as $\sigma_{e(M)}^{2}$, is nonincreasing in *M* and for this speech frame, appears to flatten out as *M* approaches the selected order M=10. The incremental mutual information gains listed in the middle column show that while this term is always greater than or equal to zero, the incremental mutual information gain is not monotonic and can have a relatively large value for higher orders. For example, for M=8 the incremental mutual information gain is 0.381 bits/symbol, which is the largest such gain since M=1. The total mutual information gain in the rightmost column is monotonically increasing and effectively flattens out at M=8 where $I(X_{N};X^{N-M})$ is 1.499 bits/symbol.

We see from Figure 2 that there are four peaks in the spectral envelope and by inspection of the table, significant changes in the incremental mutual information gain occur as the predictor order is changed from 0 to 1, 1 to 2, 3 to 4, (more subtly from) 5 to 6, and from 7 to 8. Therefore, as the increasing predictor order allows the locations and bandwidths of the spectral peaks to be captured, there are corresponding jumps in the incremental mutual information gain.

There can be considerable variation in the total mutual information gain across different frames in the same sentence spoken by the same speaker. This is evident from Table 3, wherein it is shown that three other frames in the same utterance studied in Table 2 have mutual information gains that vary over the range of 0.83 bits/symbol to 1.968 bits/symbol. This is not unusual for speech sequences; while the predictor order is related to the length of the speaker vocal tract [9,12], the best AR model order can vary based on what is being said, in general, and with the coupling of the nasal cavity, which causes an increase in the short term linear redundancy. There can also be interaction with the longer term memory due to speaker pitch, resulting in the AR model order varying considerably for the same speaker.

### 7.2. Long Term Redundancy

Of course, while an AR model is useful to capture the short term memory in a speech waveform, it is well known that there is a long term memory related to speaker pitch as well. In Figure 2, by inspection we can see that there is a longer term redundancy with a memory of roughly 50 samples. In terms of relative entropy, the longer term memory due to speaker pitch can be explicitly exhibited by breaking the term due to linear redundancy in Equation (28) into two terms, one involving short term linear redundancy (AR sequence model) and the other involving the long term redundancy due to speaker pitch as

(46) $$\begin{array}{cc} R= & D_{N}(P_{X}^{\left(N\right)}\left(X\right)\|U^{\left(N\right)})=D_{M}(P_{X}^{\left(M\right)}\left(X\right)\|\prod_{k=1}^{M}P_{X}^{\left(1\right)}\left(x_{k}\right))\\ + & D_{L}(P_{X}^{\left(L\right)}\left(X\right)\|\prod_{k=1}^{L}P_{X}^{\left(1\right)}\left(x_{k}\right))+D_{N}(\prod_{k=1}^{N}P_{X}^{\left(1\right)}\left(x_{k}\right)\|U^{\left(N\right)}) \end{array}$$

where we now have the total sequence length being analyzed as *N*, the short term AR memory as *M* as before, and the longer term memory as *L*, with N>L>M. The notation does not illustrate the notion that while L>N, the short term AR order parameters are separate from the long term linear redundancy. The terms in Equation (46) measure the short term linear redundancy, $D_{M}$, the long term linear redundancy $D_{L}$, and the distance of the i.i.d. probabilty of the sequence from the uniform distribution, $D_{N}$, after the linear redundancies have been removed.

The speech coding literature lists a wide range of SPER gains for long term prediction; for our experiments, we observe SPER values of 1 to 3 dB. In terms of mutual information gain, this range is 0.363 to 0.996 bits/symbol. Therefore, for Frame 3237 in Table 2 the long term prediction can increase the mutual information gain by these amounts to reduce the corresponding nonlinear redundancies to the range of 1.157 to 0.524 bits/symbol.

## 8. Discussion and Conclusions

The relative entropy of a sequence is decomposed into a relative entropy describing the linear redundancy and a relative entropy representing the nonlinear redundancy, which when combined capture the total redundancy in the sequence. One component of the total redundancy, called the mutual information gain, is then expanded using the chain rule for mutual information into the sum of incremental mutual information gains. These quantities are used to analyze a purely autoregressive sequence and to express the redundancies in representations of speech signals with short term and long term linear redundancies. It is shown how inaccurate autoregressive model orders or unmodeled linear redundancies become nonlinear redundancies, thus implying a misleadingly large amount of nonlinear redundancy or apparent randomness. While the minimum mean squared prediction error for autoregressive sequences is monotonically decreasing in predictor order, the incremental mutual information gain is not since it is measured with respect to a preceding lower predictor order, so incremental mutual information gain more accurately characterizes the improvement in an AR model as the predictor order is increased.

## Figures and Tables

**Table 1:** Incremental and total mutual information gain as the predictor order is increased: zero mean, unit variance Gaussian AR(10) sequence.

*M*	$\sigma_{e(M)}^{2}$	$I(X_{N};X_{k}|X_{k-1},\ldots ,X_{N-M})$	$I(X_{N};X^{N-M})$
0	1.0	0 bits/symbol	0 bits/symbol
1	0.3111	0.842	0.842
2	0.0667	1.11	1.952
3	0.0587	0.092	2.044
4	0.0385	0.304	2.348
5	0.0375	0.019	2.367
6	0.0342	0.065	2.432
7	0.0308	0.069	2.501
8	0.0308	0.0	2.501
9	0.0261	0.12	2.621
10	0.0251	0.026	2.647
0–10	0.0251	2.647	2.647

**Table 2:** Incremental and total mutual information gain as the predictor order is increased: Frame 3237, SPER=9.15dB.

*M*	$\sigma_{e(M)}^{2}$	$I(X_{N};X_{k}|X_{k-1},\ldots ,X_{N-M})$	$I(X_{N};X^{N-M})$
0	1.0	0 bits/symbol	0 bits/symbol
1	0.402	0.656	0.656
2	0.328	0.147	0.803
3	0.294	0.0795	0.883
4	0.2465	0.125	1.01
5	0.239	0.0234	1.031
6	0.2117	0.0869	1.118
7	0.212	0.0	1.118
8	0.125	0.381	1.499
9	0.1216	0.0206	1.52
10	0.1216	0.0	1.52
0–10	0.1216	1.52	1.52

**Table 3:** Total mutual information gain for 10th order predictors and corresponding signal to prediction error ratios (SPERs) for several speech frames [13].

Speech Frame No.	SPER in dB	$I(X_{N};X^{N-M})$
23	11.85	1.968 bits/symbol
3314	7.74	1.29 bits/symbol
87	5	0.83 bits/symbol

## Abbreviations

The following abbreviations are used in this manuscript:

AR	Autoregressive
AR(M)	Autoregressive of order *M*
MMSPE	Minimum mean squared prediction error
Q	Entropy (rate) power
SPER	Signal to prediction error ratio

## Appendix A. Calculating the AR Model Coefficients

For a given windowed frame of *L* input speech samples, where the windowing sets all samples outside the window to zero (equivalent to an assumption of stationarity), it is necessary to calculate the linear prediction coefficients. This is done by choosing the coefficients to minimize the sum of the squared prediction errors over the frame; that is, choose the coefficients to minimize $\sigma_{e(M)}^{2}\left(k\right)=\sum {\left[x\left(k\right)-\sum_{i=1}^{M}a_{i}x\left(k-i\right)\right]}^{2}$ [9]. Taking the partial derivatives with respect to each of the coefficients, $a_{j}$, j=1,2,…,M, and equating to zero, yields the set of linear simultaneous equations

(A1) $$\sum_{i=1}^{M}a_{i}R(|i-j|)=R\left(j\right)$$

for j=1,2,…,M, where

(A2) $$R\left(j\right)=\frac{1}{L}\sum_{k=0}^{L-j-1}x\left(k\right)x\left(k+j\right)$$

with R(j)=R(−j). In matrix notation this becomes RA=C where **R** is an *M* by *M* Toeplitz matrix of the autocorrelation terms in Equation (A2), $A={\left[a_{1},a_{2},\ldots ,a_{M}\right]}^{T}$, and C is a column vector of the autocorrelation terms R(j),j=1,2,…,M.

### Correlation Matching

It is common in signal processing applications, and specifically in speech processing and coding applications, to use what is often called the Correlation Matching method wherein we calculate the autocorrelation terms according to Equation (A2) and then specify an AR(M) model based on these autocorrelations. Thus, the model parameters, coefficients and autocorrelations, agree with the parameters for the sequence x(i),i=1,2,…,L up to order *M*, but may not agree for other autocorrelation lags [14].

Working with the AR(M) model in Equation (43), it is straightforward to develop the recursion for the autocorrelation values as

(A3) $$R\left(k\right)=\sum_{i=1}^{M}a_{i}R(|k-i|)=a_{1}R\left(k-1\right)+a_{2}R\left(k-2\right)+\ldots +a_{M}R\left(k-M\right)$$

for all k>0. This allows us to extend the autocorrelation function to all possible lags using the M+1 estimated autocorrelations. The extended autocorrelation values will equal the true values only if the sequence is actually AR(M). Of course, most sequences, even though partially well represented in some sense using AR models, are not purely AR, and so the higher order autocorrelations will not satisfy Equation (A3).
